# Effect of Ti Addition on the Microstructure and Mechanical Properties of SiC Matrix Composites Infiltrated by Al–Si (10 wt.%)–xTi Alloy

**DOI:** 10.3390/ma12020318

**Published:** 2019-01-21

**Authors:** Yajun Yu, An Du, Xue Zhao, Yongzhe Fan, Ruina Ma, Shijie Li, Wei Wang, Yaqi Cui, Xiaoming Cao

**Affiliations:** School of Materials Science and Engineering, Hebei University of Technology, Tianjin 300130, China; yyj621@126.com(Y.Y.); duan@hebut.edu.cn(A.D.); zhaoxue_smile@163.com(X.Z.); fyz@hebut.edu.cn(Y.F.); jiayouwei99@yahoo.com(W.W.); cuiyaqiwangyi@163.com(Y.C.); gd-sam@21cn.com(X.C.)

**Keywords:** infiltration, Ti addition, Ti_3_Si(Al)C_2_, bending strength, fracture toughness

## Abstract

This paper proposes a simple reactive melt infiltration process to improve the mechanical properties of silicon carbide (SiC) ceramics. SiC matrix composites were infiltrated by Al–Si (10 wt.%)–xTi melts at 900 °C for 4 h. The effects of Ti addition on the microstructure and mechanical properties of the composites were investigated. The results showed that the three-point bending strength, fracture toughness (by single-edge notched beam test), and fracture toughness (by Vickers indentation method) of the SiC ceramics increased most by 34.3%, 48.5%, and 128.5%, respectively, following an infiltration with the Al–Si (10 wt.%)–Ti (15 wt.%) melt. A distinct white reaction layer mainly containing a Ti_3_Si(Al)C_2_ phase was formed on the surface of the composites infiltrated by Al alloys containing Ti. Ti–Al intermetallic compounds were scattered in the inner regions of the composites. With the increase in the Ti content (from 0 to 15 wt.%) in the Al alloy, the relative contents of Ti_3_Si(Al)C_2_ and Ti–Al intermetallic compounds increased. Compared with the fabricated composite infiltrated by an Al alloy without Ti, the fabricated composites infiltrated by Al alloys containing Ti showed improved overall mechanical properties owing to formation of higher relative content Ti_3_Si(Al)C_2_ phase and small amounts of Ti–Al intermetallic compounds.

## 1. Introduction

Silicon carbide (SiC) ceramics are promising materials for applications in various industries owing to their high thermal and chemical stability, good resistance to corrosion and thermal shock, and high strength. However, their extensive application is limited due to their poor toughness [1,2,3]. To improve the strength and toughness of SiC ceramics, processing techniques, such as chemical vapor infiltration (CVI), liquid polymer infiltration, reactive melt infiltration (RMI), and spark plasma sintering (SPS), were applied [4,5,6]. Among these methods, RMI, which is most suitable for realizing the net shape of SiC matrix composites at low cost, was found to satisfy the above requirements [7]. During RMI, a molten metal (such as an aluminum alloy) spontaneously infiltrates the pores driven by the capillary force and reacts with the ceramic to form bulk compounds [8].

A good wettability between the SiC matrix and the aluminum alloy is essential for RMI. Unfortunately, a pure aluminum melt cannot wet SiC below 900 °C [9]. Although the wettability can be improved at higher temperatures, the interfacial reaction, i.e., 4Al + 3SiC = Al_4_C_3_ + 3Si, easily occurs at temperatures above 900 °C, thus degrading the strength, modulus, and corrosion resistance of the composites under service conditions because of the hygroscopic behavior of the reaction products (Al_4_C_3_) [10,11]. To improve the wettability and avoid the formation of Al_4_C_3_, the addition of elemental Si and Ti was proposed [12,13,14,15,16]. Adding Ti to the Al–Si system results in a new Ti_3_Si(Al)C_2_ phase in situ in the SiC matrix; this is a novel structural material having a high toughness because of the combination of the ceramic and metallic properties [17,18,19]. Ti_3_Si(Al)C_2_ can be combined with SiC to improve the overall performance of the material through the RMI, making it suitable for use under complex service conditions [20].

In this work, SiC ceramics were modified by applying RMI with aluminum alloys at atmospheric pressure. The effect of adding Ti to the Al–Si alloy (Si: 10 wt.%) on the microstructure and mechanical properties of the SiC matrix composites was investigated. The phase compositions of the samples were characterized using scanning electron microscopy (SEM) and X-ray diffraction (XRD). The mechanical properties were measured using a universal testing machine and a Vickers indenter.

## 2. Materials and Methods 

### 2.1. Material Preparation

The commercial SiC ceramic (3.02 g/cm^3^, Huamei Ceramics Co., Ltd., Weifang, China) was composed of SiC and Si phases. It was prepared by reactive sintering at about 1750 °C by high-temperature siliconizing. The relative volume fraction of Si was 16%. The open porosity of the SiC ceramic was 1.48%. It was used as a substrate material for infiltration. Before the infiltration, the SiC ceramic was cut into specimens with dimensions of 40 mm × 5 mm × 5 mm and 36 mm × 4 mm ×2 mm using electro-discharge machining. The surfaces of the composites were successively ground with 400-, 800-, 1000-, and 1500-grit diamond grinding papers and then ultrasonically cleaned in acetone and alcohol for 8 min each. An aluminum alloy was obtained by smelting an aluminum ingot (99.5%; Fuxin Jinshu Co., Ltd., Baoji, China), titanium ingot (99.9%; Fuxin Jinshu Co., Ltd., Baoji, China), and silicon piece (99.5%; PRAM Co., Ltd., Beijing, China) in a vacuum furnace. Four types of aluminum alloys were used. Table 1 lists their chemical compositions. The prepared Al alloy was placed in a crucible and melted in an electric furnace. Subsequently, the SiC ceramics were dipped into the melt and heated at 900 °C for 4 h in the same electric furnace to provide conditions for the infiltration.

### 2.2. Microstructure and Phase Composition

The polished cross-sectional surfaces and fracture surfaces were observed using a scanning electron microscope equipped with an energy-dispersive spectroscopy (EDS; Nova Nano SEM450, FEI Co., Hillsboro, OR, USA) system. The phases were identified via X-ray diffraction (Advance D8 diffractometer, Bruker AXS Co., Karlsruhe, Germany) with Cu Kα radiation at 40 kV and 40 mA. The data were digitally recorded in a continuous scanning mode for angles (2θ) ranging from 10° to 90° with a scanning rate of 10°·min^−1^.

### 2.3. Mechanical Properties

Small bars with dimensions of 40 mm × 5 mm × 5 mm were machined for the bending strength tests and the Vickers hardness tests. Small bars with dimensions of 36 mm × 4 mm × 2 mm (*b* = 2 mm and *w* = 4 mm) were machined for fracture toughness tests. The Vickers hardness was measured using a Vickers indenter (AKASHI AVK-A, Shimadzu, Kyoto, Japan) at a load of 4.9 N for 15 s. The results of at least 15 individual tests were averaged for the final reading. The bending strength and fracture toughness were measured by conducting three-point bending tests and single-edge notched beam (SENB) tests (with a 0.02 mm (width) × 2 mm (depth) crack) on a CMT6104 universal testing machine (Xinsansi Co., Shenzhen, China) at cross-head speeds of 0.5 and 0.05 mm/min with loading spans of 30 and 20 mm, respectively. Ten bars of each sample were tested, and the average value was determined. The fracture toughness was then calculated using the following equations [21]:

(1) $$K_{IC}=\left(PL/bw^{3/2}\right)f\left(a/w\right)$$

(2) $$f\left(\frac{a}{w}\right)=\frac{3{\left(a/w\right)}^{1/2}\left[1.99-\left(a/w\right)\left(1-a/w\right)\left(2.15-3.93a/w+2.7a^{2}/w^{2}\right)\right]}{2\left(1+2a/w\right){\left(1-a/w\right)}^{3/2}}$$

where *K_IC_* is the fracture toughness, *P* and *L* are the fracture load and lower span, respectively, *b* and *w* are the width and thickness of the specimen, respectively, and *a* is the depth of the notch.

The fracture toughness of the samples was also measured using the Vickers indentation method to study the toughening mechanism of Ti_3_Si(Al)C_2_. From the indentations and developed cracks, an estimate of the fracture toughness of the samples was made according to the following equation contrived by Anstis et al. [22]:

(3) $$K_{IC}=0.016{\left(\frac{E}{H}\right)}^{0.5}\times \left(\frac{P}{c^{3/2}}\right)\times 10^{-6}$$

where *H* is the Vickers hardness in MPa, *P* is the indentation load in N, *E* is Young’s modulus in MPa, and *c* is the distance from the center of the indentation to the extremity of the crack in meters.

## 3. Results and Discussion

### 3.1. Phase Composition, Microstructure, and Infiltration Process

**Figure 1:**
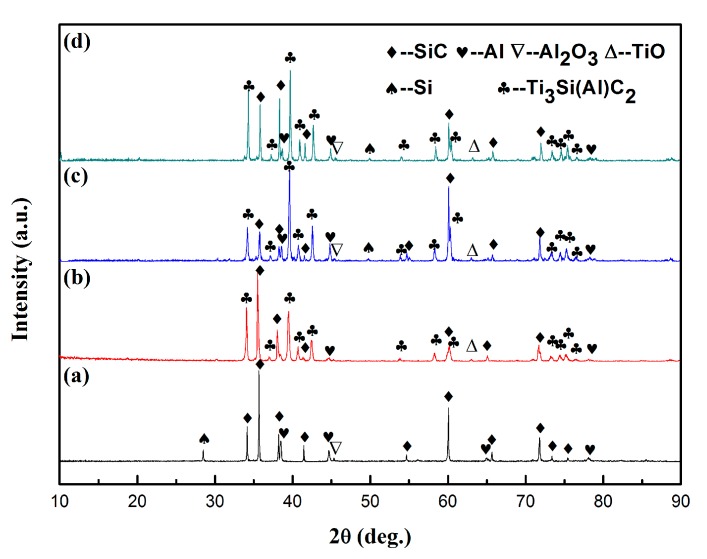
X-ray diffraction (XRD) patterns on the surface of the composites infiltrated by the Al–Si–xTi alloy (Si: 10 wt.%) with Ti contents (wt.%) of (**a**) 0, (**b**) 5, (**c**) 10, and (**d**) 15.

Figure 1 shows the X-ray diffraction patterns on the surface of the composites infiltrated by the Al–Si–xTi alloy (Si: 10 wt.%) with Ti contents varying from 0 to 15 wt.%. Without Ti addition, the composites were composed of SiC, Al, and Si infiltrated by the Al alloy, whereas the composites containing Ti were composed of Ti_3_Si(Al)C_2_ as the main phase with small amounts of SiC and C. This result indicates that the introduction of Ti is beneficial to the formation of Ti_3_Si(Al)C_2_. With the increase in the Ti content, the diffraction peaks of Ti_3_Si(Al)C_2_ became stronger, indicating the increase in the relative content of Ti_3_Si(Al)C_2_.

**Figure 2:**
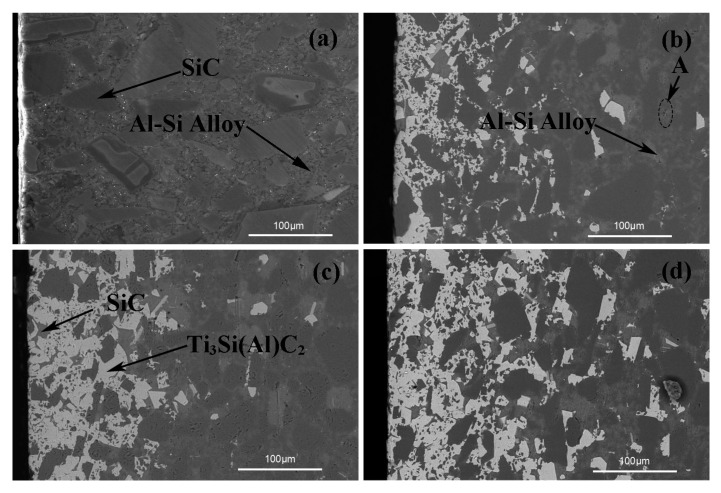
Cross-sectional backscattered electron (BSE) images of the reaction layers on the composites infiltrated by the Al–Si–xTi alloy (Si: 10 wt.%) with Ti contents (wt.%) of (**a**) 0, (**b**) 5, (**c**) 10, and (**d**) 15.

Figure 2 shows the cross-sectional backscattered electron (BSE) images of the composites infiltrated by the Al–Si–xTi alloy (Si: 10 wt.%; x = 0, 5, 10, and 15 wt.%). Table 2 lists the EDS analysis results of the different regions shown in Figure 2. As shown in Figure 2b–d, a distinct white reaction layer was formed on the surface of each composite infiltrated by the Al–Si–xTi alloy containing Ti. In contrast, there was no obvious reaction layer on the surface of the composite infiltrated by the Al–Si alloy (Si: 10 wt.%) (Figure 2a). Based on the EDS analysis results (listed in Table 2) and XRD patterns (shown in Figure 1), the white reaction layer is suggested to be Ti_3_Si(Al)C_2_ with a small amount of SiC. The dark phase indicates SiC, and the gray phases comprising elements slightly heavier than SiC are considered to be Al–Si with a small amount of C. Therefore, the point A (shown in Figure 2b) indicates the Al–Si phase. The white reaction layer containing Ti_3_Si(Al)C_2_ gets thicker as we move from Figure 2b–d. The maximum thickness of the relatively dense white reaction layer was approximately 110 µm in the composites infiltrated by the Al–Si (10 wt.%)–Ti (15 wt.%) alloy. The thickness of the less dense white reaction layers was approximately in the range of 0.3–0.4 mm. This shows that increasing the Ti content in the Al alloy significantly increased the relative content of Ti_3_Si(Al)C_2_ formed near the surface of the composites; this is consistent with the XRD results.

Based on the EDS analysis results (listed in Table 2) and the BSE images (shown in Figure 2), we find that most of the aluminum seems to exist in the Al–Si phase with a small amount of C in the gaps between the SiC particles. Compared with the porosity of prefabricated materials (10–60 vol.%) used in conventional melt infiltration methods, the porosity of the SiC ceramics employed in this study was low (1.48 vol.%). An aluminum alloy can infiltrate an SiC ceramic, mainly because the contact between the aluminum alloy and Si in the SiC matrix generates a low-melting liquid Al–Si alloy at the position of Si in the matrix; this alloy infiltrates into the SiC ceramic along the position of Si in the matrix. The relative volume fraction of Si was 16%. This provided channels for the Al alloy to infiltrate the SiC ceramics, unlike that observed in the conventional penetration of porous prefabricated parts under the action of a capillary force.

The mechanisms of the interaction of the melt with the SiC substrate in the form of possible reactions that take place can be summarized. When a liquid Al alloy melt infiltrates the SiC ceramics, the SiC ceramics undergo wetting, infiltration, and a reaction process because of the Al alloy melt. Firstly, the Al alloy directly contacts SiC and then rapidly penetrates the SiC ceramic owing to the good wettability between them. The melted Al alloy dissolves free Si in the SiC substrate while occupying the original position of free Si, resulting in the surrounding of the SiC particles. An Al–Si eutectic alloy is formed from a possible reaction mechanism (Equation (4)). Meanwhile, Ti in the Al alloy melt rapidly accumulates and adsorbs around the SiC particles near the surface. Based on the initial composition and Ti–Si–C ternary phase diagram, multiple reaction paths are possible for the Ti_3_Si(Al)C_2_ synthesis via the infiltration method, as described below. In this study, we propose a possible reaction mechanism for the formation of Ti_3_Si(Al)C_2_ through the direct reaction between SiC, Si, and Al in the melt (Equation (5)). However, most of the Ti is adsorbed onto the surface of the SiC ceramic samples, which is consistent with a previous study [23]. With the increase in the Ti content in the Al alloy, the SiC ceramic matrix can be efficiently wetted by the liquid Al–Si–xTi alloy (Si: 10 wt.%). Increasing the Ti content can promote the reaction between Ti in the Al–Si eutectic alloy and the matrix [24]. Meanwhile, a small amount of Ti infiltrated the ceramic with Al alloy, forming some possible Ti–Al intermetallic compounds. It can be inferred that Ti in the aluminum alloy helps reduce the surface tension of the alloy at 900 °C. These results are consistent with those obtained by Narciso [25].

(4) $$\mathrm{Al}+\mathrm{Si}\to \mathrm{Al}-{\mathrm{Si}}_{\mathrm{eutectic}}$$

(5) $$2\mathrm{SiC}+3\mathrm{Ti}+\mathrm{Al}\to {\mathrm{Ti}}_{3}\mathrm{Si}\left(\mathrm{Al}\right)C_{2}+\mathrm{Si}$$

**Figure 3:**
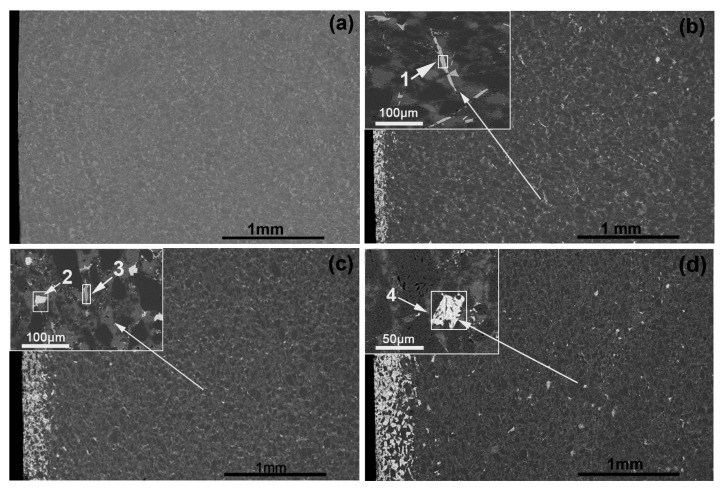
Low-magnification cross-sectional BSE images of the reaction layers and inner regions of the composites infiltrated by the Al–Si–xTi alloy (Si: 10 wt.%) with Ti contents (wt.%) of (**a**) 0, (**b**) 5, (**c**) 10, and (**d**) 15.

To further study the distribution of Ti, the cross-sections of the specimens after infiltration were analyzed in detail. Figure 3 shows the low-magnification cross-sectional BSE images of the reaction layers and inner regions of the composites infiltrated by the Al–Si–xTi alloy (Si: 10 wt.%). Table 3 lists the EDS analysis results of the different regions shown in Figure 3. Some light-white phases can be found in the inner regions of the composites containing Ti, as shown in Figure 3b–d. These light-white phases were scattered throughout the inner regions. Based on the EDS analysis of regions 1 and 3, the atomic ratio of Ti to Al was approximately 1:3. Therefore, the long strip phase in these regions is suggested to be TiAl_3_. The atomic ratio of Ti to Al in regions 2 and 4 was approximately 3:1. Therefore, the irregular, block-shaped white phase in these regions is suggested to be Ti_3_Al, which is another type of Ti–Al intermetallic compound. This shows that increasing the Ti content in the Al alloy promoted the formation of new Ti–Al intermetallic compounds. These compounds were scattered in the inner regions of the composites containing Ti. Based on the composition and Ti–Al binary phase diagram, we propose two possible reaction mechanisms for the formation of Ti_3_Al and TiAl_3_ through the direct reaction between Al and Ti in the melt (Equations (6) and (7)).

(6) $$3\mathrm{Al}+\mathrm{Ti}\to {\mathrm{TiAl}}_{3}$$

(7) $$\mathrm{Al}+3\mathrm{Ti}\to {\mathrm{Ti}}_{3}\mathrm{Al}$$

### 3.2. Mechanical Properties

**Figure 4:**
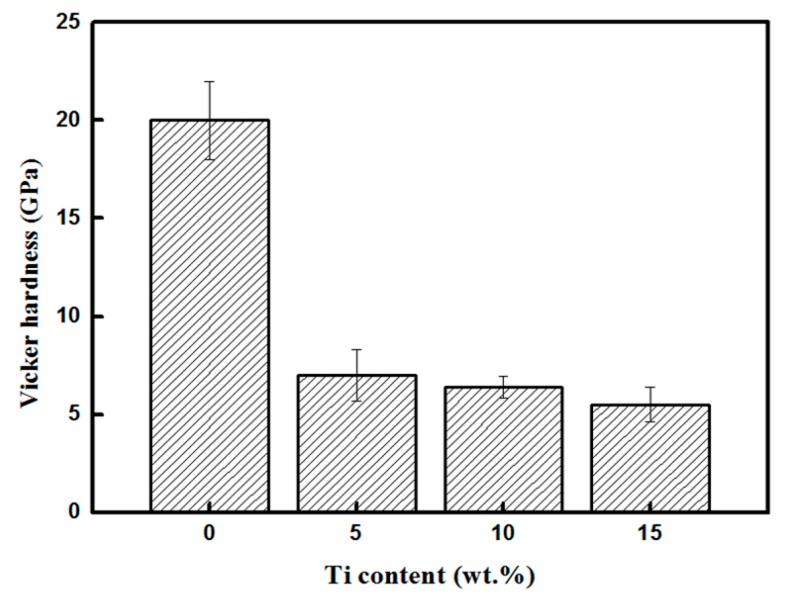
Vickers hardness values of the composites infiltrated by the Al–Si–xTi alloy (Si: 10 wt.%).

Figure 4 shows the results of the Vickers hardness test conducted on the composites infiltrated by the Al–Si–xTi alloy (Si: 10 wt.%). The hardness value of the composite infiltrated by the Al–Si alloy (Si: 10 wt.%) was approximately 20 GPa, which was significantly higher than that of the composites containing Ti. The Vickers hardness decreased with the increase in the Ti content. This is mainly because the composite infiltrated by the Al–Si alloy was largely composed of SiC, whereas the ones containing Ti exhibited Ti_3_Si(Al)C_2_ on the surface, the hardness of which is lower than that of SiC.

**Figure 5:**
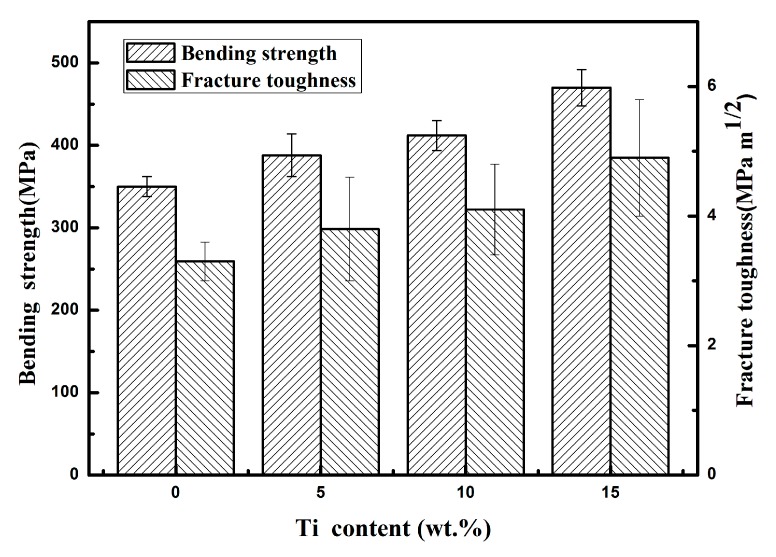
Bending strength and fracture toughness (determined using the single-edge notched beam (SENB) test) of the composites infiltrated by the Al–Si–xTi alloy (Si: 10 wt.%).

Figure 5 shows the bending strength and fracture toughness (determined using SENB test) of the composites with and without Ti content. Table 4 lists the fracture toughness values of the composites measured using the Vickers indentation method (VIM). With the increase in the Ti content, the bending strength of the composites increased from 350 to 470 MPa and the fracture toughness (determined using SENB test) increased from 3.30 to 4.90 MPa·m^1/2^. However, the fracture toughness (determined using VIM) increased from 2.87 MPa·m^1/2^ to 6.56 MPa·m^1/2^. The fracture toughness values of the composites containing Ti obtained from the VIM method were higher than those obtained from the SENB test because the content of Ti_3_Si(Al)C_2_ formed near the surface is high. The toughness values of Ti_3_Si(Al)C_2_ were reported to range from 5 to 20 MPa·m^1/2^. The bending strength and fracture toughness of the SiC substrate were 185 MPa [17] and 1.7 MPa·m^1/2^ (tested in this work), respectively, which are not shown in Figure 5. The bending strength and fracture toughness (determined using SENB test) of the SiC ceramics increased by 89 and 94%, respectively, after infiltration with the Al–Si alloy (Si: 10 wt.%). The improvement in the mechanical properties was attributed to the complete infiltration with the Al alloy. In other words, the aluminum alloy infiltrated the inner regions of the samples evenly. The Al–Si alloy was a relatively soft, ductile phase compared with the hard, brittle SiC and Si phases. Therefore, the presence of the Al–Si alloy in the four fabricated composites helped improve the overall properties of the SiC matrix. In our previous study, we easily achieved complete infiltration with the Al alloy after infiltrating with the Al–Si alloy (Si: 10 wt.%) for 4 h [17]. In this work, the Al–Si–xTi alloy (Si: 10 wt.%) was used for the infiltration. The Al alloy completely penetrated the inner regions of all the four fabricated composites. The Al–Si content in the four composites was largely the same. As a result, the effect of the Al–Si alloy on the performance of each composite was considered identical. As such, we focused only on the effect of the Ti content in the Al alloy. The improved bending strength and fracture toughness could be attributed to the formation of the Ti-rich surface layer mainly containing Ti_3_Si(Al)C_2_. As shown in Figure 3, the TiAl_3_ and Ti_3_Al phases were formed in the inner regions of the three composites. The fracture toughness value of Ti_3_Al was reported to be 10 MPa·m^1/2^ [26], which is approximately six times that of the original SiC. Moreover, the fracture toughness value of TiAl_3_ is higher than that of SiC. Although the Ti-containing phases are not as dense as the surface reaction layer, they do play a certain role in improving the ductility of the composites. Therefore, the formation of the Ti–Al intermetallic compounds scattered in the inner regions could also help enhance the overall mechanical properties. To determine their specific mechanism, we need to further study the microstructure of the composite fracture.

**Figure 6:**
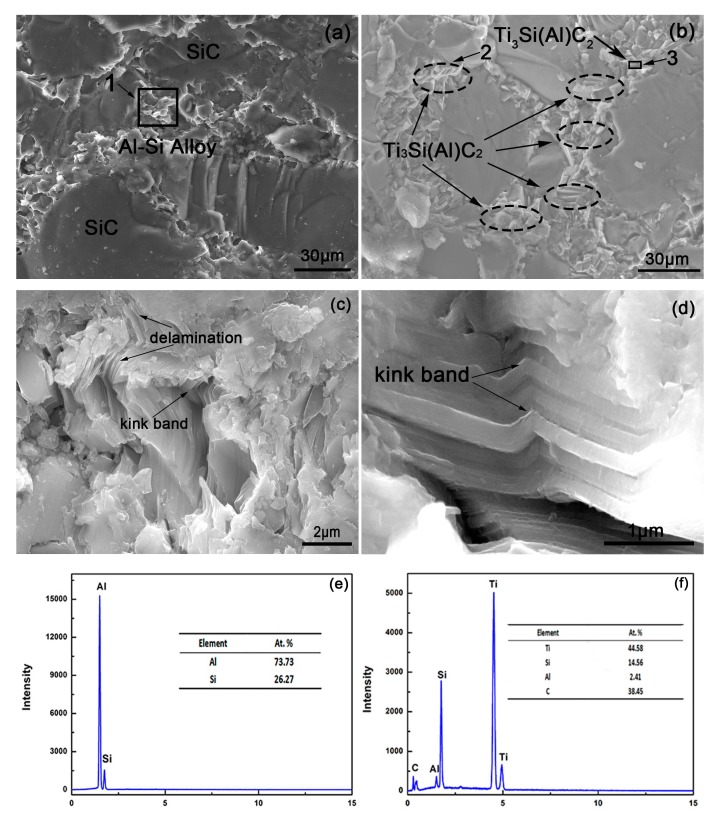
SEM micrographs of the fracture surface of the composites infiltrated by the Al–Si–xTi alloy (Si: 10 wt.%): (**a**) x = 0 wt.%; (**b**) x = 15 wt.%. (**c**) Higher-magnification microstructure of region 2 shown in Figure 6b; (**d**) higher-magnification microstructure of region 3 shown in Figure 6b. (**e**) EDS analysis of region 1 shown in Figure 6a; (**f**) EDS analysis of Ti_3_Si(Al)C_2_ shown in Figure 6b.

Figure 6a,b show the fracture surfaces of the composites without and with Ti. Although some white ductile Ti–Al intermetallic compounds were scattered in the inner regions of the composites infiltrated by the Al alloy with Ti addition, shown in Figure 3, their fracture micrographs were too difficult to obtain because of their low content. Therefore, the main difference between the three composites infiltrated by the Al alloy with different Ti contents was the content of Ti_3_Si(Al)C_2_ formed near the surface. Thus, the composite infiltrated by the Al–Si (10 wt.%)–Ti (15 wt.%) alloy was typical enough to illustrate the mechanisms of Ti_3_Si(Al)C_2_. Some white particles were distributed in the gaps of the composites surrounding the SiC; these were the Al and Al–Si eutectic phases. With the increase in the Ti content, Ti_3_Si(Al)C_2_ sliced with different thicknesses around SiC can be easily identified in Figure 6b.

Figure 6c shows a higher-magnification image of region 2 shown in Figure 6b. Figure 6d is a higher-magnification of region 3 in Figure 6b. Slice shapes of Ti_3_Si(Al)C_2_ are obvious in regions 2 and 3, confirmed by the EDS analysis shown in Figure 6f. In the loading process, deformation modes of delamination and kink band occur to promote the crack deflection, which absorbs a considerable amount of energy and helps improve the bending strength [18]. These energy-absorbing mechanisms of Ti_3_Si(Al)C_2_ suggest that the fabricated composites infiltrated by the Al alloy with Ti exhibited some ductility. The fabricated composite infiltrated by the Al–Si (10 wt.%)–Ti (15 wt.%) alloy had the highest thickness of the reaction layer containing Ti_3_Si(Al)C_2_. Moreover, the formation of TiAl_3_ and Ti_3_Al in the inner regions played a certain role in improving the toughness of the composites. The stronger grain interfaces may be another reason for the toughening because of the reactivity of Ti with SiC which reduced intergranular cracking (Figure 6). Therefore, the three-point bending strength and fracture toughness of the SiC matrix composites infiltrated by the Al–Si (10 wt.%)–Ti (15 wt.%) melt increased most by 34.3 and 48.5%, respectively, compared with those of the SiC matrix composites infiltrated by the Al–Si melt (Si: 10 wt.%).

**Figure 7:**
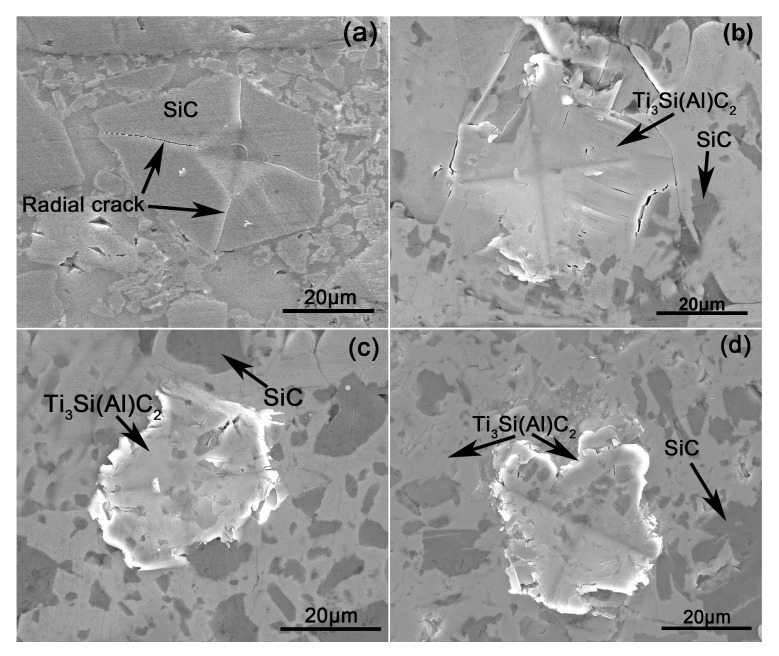
Indentation micrographs of the polished surfaces of the composites infiltrated by the Al–Si–xTi alloy (Si: 10 wt.%) with Ti contents (wt.%) of (**a**) 0, (**b**) 5, (**c**) 10, and (**d**) 15.

**Figure 8:**
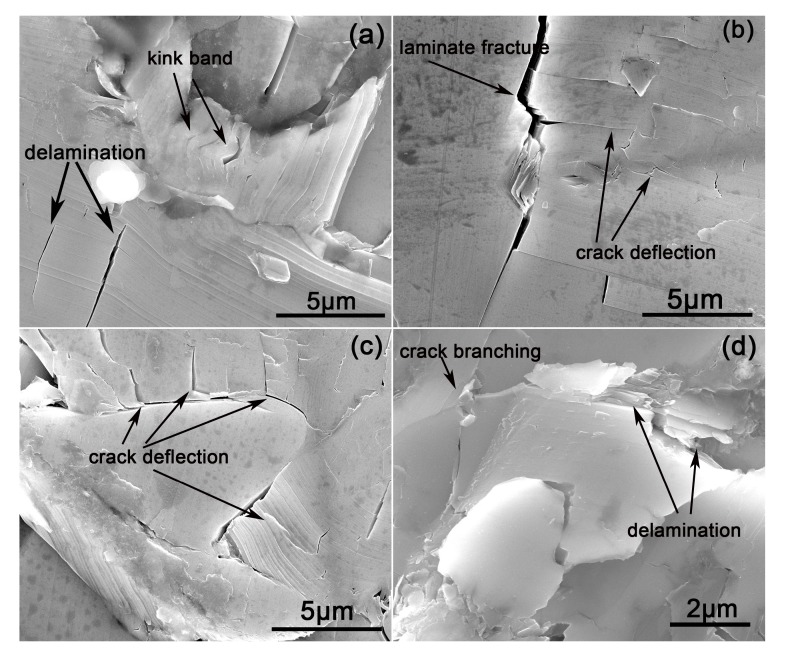
Energy-absorbing mechanisms in Ti_3_Si(Al)C_2_: (**a**) delamination and kink-band formation; (**b**) laminate fracture and crack deflection; (**c**) crack deflection; (**d**) crack branching and delamination.

The relationship between the microstructure and the toughening mechanism of the reaction layers on the fabricated composites was further clarified from the crack propagation paths in the typical phase obtained using the indentation method (Figure 7). Figure 8 shows the energy-absorbing mechanisms of Ti_3_Si(Al)C_2_ shown in Figure 7. Figure 7a–d show the indentation micrographs of the polished surfaces of the composites infiltrated by the Al–Si–xTi alloy (Si: 10 wt.%) (x = 0, 5, 10, and 15 wt.%); the experiment was carried out at a load of 4.9 N. The areas affected by the indentation in the composites infiltrated by the Al–Si alloy (Si: 10 wt.%) (Figure 7a) were obviously large, and the cracks propagated diagonally along the ends of the indentation to a large extent. On the paths of crack propagation, the cracks passed directly through the SiC grain, leading to four radial cracks. On the other hand, the damage areas of the composites infiltrated by the alloys containing Ti (Figure 7b–d) were confined to the vicinity of the indentation, and the crack propagation from the corners was limited. The mechanisms of energy absorption of Ti_3_Si(Al)C_2_ (from Figure 7b–d) can be clearly observed in the higher-magnification SEM micrographs shown in Figure 8. The delamination and kink-band formation can be observed in Figure 8a. The laminate fracture and crack deflection can be observed in Figure 8b,c. Crack branching and delamination can be observed on the fracture surfaces shown in Figure 8d. The paths of crack propagation consume the fracture energy, which is beneficial to the improvement of the surface fracture toughness and strength of the composites. This suggests a high resistance to damage and correspondingly high toughness of the composites containing Ti_3_Si(Al)C_2_. Moreover, the resistance to crack propagation in the composites infiltrated by the alloys containing Ti increased with the increase in the Ti content because of the formation of relatively higher content Ti_3_Si(Al)C_2_ near the surface.

## 4. Conclusions

In this paper, SiC matrix composites were infiltrated by Al–Si–xTi alloys (Si: 10 wt.%) (x = 0, 5, 10, and 15 wt.%) at 900 °C for 4 h. The effects of Ti on the microstructure and mechanical properties of the SiC matrix composites were investigated. With the increase in the Ti content from 0 to 15 wt.%, the thickness of the reaction layer mainly containing the Ti_3_Si(Al)C_2_ phase increased. The maximum thickness reached up to 110 μm. The three-point bending strength and the fracture toughness—determined by performing the single-edge notched beam test and the Vickers indentation method—of the SiC matrix composites infiltrated by the Al–Si (10 wt.%)–Ti (15 wt.%) melt increased by 34.3%, 48.5%, and 128.5%, respectively, compared with those of the SiC matrix composites infiltrated by the Al–Si melt. The maximum bending strength and fracture toughness of the composites prepared with 15 wt.% Ti reached 470 MPa and 4.90 MPa·m^1/2^, respectively. Moreover, the fracture toughness (determined using the Vickers indentation method) of the composites fabricated reached 6.56 MPa·m^1/2^. The work demonstrated that the enhancement in the fracture toughness of the overall SiC matrix materials infiltrated by alloys containing Ti could be attributed to the formation of Ti–Al intermetallics in the inner regions, all of which were more ductile than SiC and Si. Moreover, the improvements in the three-point bending strength and fracture toughness of the surface could be mainly attributed to the formation of higher relative content Ti_3_Si(Al)C_2_ near the surface.

## Figures and Tables

**Table 1:** Ingredients of various alloys.

Alloying Content (wt.%)				
Al–Si (10 wt.%)–xTi Alloy	x = 0	x = 5	x = 10	x = 15
Ti	0	5	10	15
Si	10	10	10	10
Al	90	85	80	75

**Table 2:** Energy-dispersive spectroscopy (EDS) analysis results of the different regions in the interfacial reaction layer shown in Figure 2.

Region	AlK (at.%)	SiK (at.%)	CK (at.%)	TiK (at.%)
SiC	1.66	39.32	58.20	0.82
Ti_3_Si(Al)C_2_	6.79	16.43	30.40	46.38
A	62.82	25.75	11.43	-

**Table 3:** EDS analysis results of different inner regions shown in Figure 3.

Region	AlK (at.%)	SiK (at.%)	CK (at.%)	TiK (at.%)
1	48.07	10.91	22.79	18.23
2	11.22	51.26	7.54	29.98
3	62.77	13.60	2.17	21.46
4	11.94	50.88	6.68	30.50

**Table 4:** Fracture toughness values of the composites infiltrated by the Al–Si–xTi alloy (Si: 10 wt.%), measured using the Vickers indentation method.

Composites Infiltrated by Al–Si–xTi Alloy (Si: 10 wt.%)	x = 0	x = 5	x = 10	x = 15
K_IC_ (MPa·m^1/2^)	2.87	5.78	6.16	6.56
